# The Role of the Family in Alcohol Use Disorder Recovery for Adults

**DOI:** 10.35946/arcr.v41.1.06

**Published:** 2021-05-06

**Authors:** Barbara S. McCrady, Julianne C. Flanagan

**Affiliations:** 1 Department of Psychology and Center on Alcoholism, Substance Abuse, and Addictions, University of New Mexico, Albuquerque, New Mexico; 2 Department of Psychiatry and Behavioral Sciences, College of Medicine, Medical University of South Carolina, Charleston, South Carolina; 3 Ralph H. Johnson Veterans Affairs Medical Center, Charleston, South Carolina

**Keywords:** alcohol, adult, alcohol treatment, couples, family therapy, recovery

## Abstract

Alcohol use disorder (AUD) and family functioning are inextricably bound, and families are impacted negatively by AUD, but families show substantial improvements with AUD recovery. Family members can successfully motivate a person with AUD to initiate changes in drinking or to seek AUD treatment. During recovery, family members can provide active support for recovery. Several couple- or family-involved treatments for AUD have been developed and tested in rigorous efficacy trials. Efficacious treatments based in family systems theory or cognitive behavioral approaches focus on the concerned family member alone, or they engage the couple or family as a unit in the treatment. However, most treatments have been studied in fairly homogeneous, heterosexual, White, non-Hispanic populations, limiting the potential generalizability of these treatments. Substantial gaps remain in our understanding of family processes associated with the initiation and maintenance of AUD recovery among adults. This review outlines the existing literature and describes opportunities for future research to address knowledge gaps in understanding the mechanisms by which these treatments are efficacious, use of family-based treatments with diverse populations, integration of pharmacotherapies with family-involved treatment, role of families in recovery-oriented systems of care, and how to improve treatment development and dissemination.

It is almost axiomatic that alcohol use disorder (AUD) and the family are inextricably bound. AUD harms individual family members and the functioning of the family as a whole, and family members’ actions may exacerbate problematic drinking. Conversely, families play a key role in recovery from AUD, and recovery has a positive impact on family members and family functioning. Scientific research to understand the interrelationships between drinking and family functioning began in the early 1900s, and treatment models that address both drinking and family functioning have been developed and tested for close to 75 years. This article reviews the conceptual and empirical literature on the impact of AUD on families, the role of the family in recovery from AUD, the role of family-involved treatment in fostering recovery, and issues related to specific populations. The review concludes with suggested future directions for research. When discussing families, we are using the term broadly to refer to a broad range of kinship relationships. When discussing couples, we are referring to couples in intimate relationships regardless of marital or co-habiting status, and using the term “partner” to refer to either individual in the intimate relationship. However, where research findings apply to a more limited group (e.g., spouse versus partner) we use the correct term to delimit the population studied. Given the limitations of current research findings, we are referring to different-sex couples unless otherwise specified.

## THE IMPACT OF AUD ON FAMILIES

AUD affects the functioning of families: Family members take on additional household and childcare responsibilities, social events are disrupted, and families may experience significant financial difficulties.^1^ Individual members of these families suffer as well. Spouses and children of adults with AUD or other substance use disorder (SUD). experience psychological distress as well as health and behavioral problems. For example, women with a male partner who has AUD and is actively drinking reported elevated levels of depression, anxiety and psychosomatic complaints, and disruptions to work and social/leisure activities, and they utilize more health care resources.^2^–^4^ Similarly, children who have a parent with AUD experience a variety of psychological, behavioral, and school problems.^5^,^6^

Research also has demonstrated a reciprocal relationship between drinking, AUD, and the quality of intimate relationships. For example, longitudinal studies of engaged different-sex couples have found that the husband’s drinking prior to marriage is a strong predictor of the wife’s drinking a year into marriage,^7^ that the female partner’s drinking influences the male partner’s drinking in the next year,^8^ and that relationship distress and AUD are strongly related.^9^ A recent meta-analysis of 17 studies (*N* = 10,553 couples) focused on different-sex couples found that partners influence one another’s drinking, although the magnitude of effects was modest. The extent to which women influenced men’s drinking (β = .19) was slightly greater than the extent to which men influenced women’s drinking (β = .12).^8^ Results from clinical and nonclinical samples also reveal a close association between heavy drinking and the perpetration of intimate partner violence.^10^ Couples with at least one partner with AUD have high rates of intimate partner violence, regardless of the sex of the partner with AUD,^11^ and drinking is common during episodes of interpersonal violence.^12^ Most typically, interpersonal violence is bidirectional in these couples.

Orford and his colleagues have proposed that the functioning of family members of those with AUD is best understood within a stress-strain-coping-support (SSCS) framework.^13^ The SSCS model assumes that living with a family member with AUD is a stressful circumstance, putting family members at risk of a variety of psychological and physical health problems. Within this model, families are seen as engaging in a variety of behaviors to cope with this chronic stressor, some of which are more effective in helping families to cope with and to influence the drinker’s behavior, and others that are less effective. The SSCS framework has informed much of contemporary research on AUD and the family.

## THE ROLE OF THE FAMILY IN RECOVERY FROM AUD

There are strong connections between family functioning and drinking outcomes. Family behaviors can contribute to changes in drinking, and, conversely, changes in drinking can contribute to more positive family functioning. For example, in early studies, Moos and colleagues examined the longitudinal course of functioning in families of men receiving treatment for AUD. At 2-year follow-up, they compared family functioning for men who were in recovery to men who had relapsed. Wives of men in recovery, compared to wives of men who relapsed, drank less, were less depressed and anxious, had fewer negative life events, and had higher family incomes.^14^ Similarly, the children of the men in recovery showed fewer symptoms of emotional distress.^15^ As a whole, families of men in recovery had greater family cohesion, greater expressiveness, a higher orientation toward recreational activities, and greater agreement in how they viewed the overall environment of their families, compared to families of men who had relapsed.^16^ These studies highlight the positive impact of recovery on families.

Families may play a key role in fostering the initiation of recovery. Although popular literature and 12-step mutual help groups for families, such as Al-Anon (https://al-anon.org/), emphasize detachment for family members and empirically supported interventions for families, such as Community Reinforcement and Family Training (CRAFT),^17^ it has been found that family behavior can increase the probability that an individual will seek help for AUD.^18^ Key family behaviors that support the initiation of change include ignoring behaviors associated with using alcohol or drugs, reinforcing positive or desirable behaviors related to sobriety or help-seeking, allowing the drinker to experience the naturally occurring negative consequences of drinking, and making specific and positive requests for changes in behavior related to drinking, such as reducing consumption or seeking help.^17^

Families and other members of the social network of persons with AUD also play an important role in supporting successful changes in drinking.^19^ Although the scientific literature is limited on specific family behaviors that facilitate and support successful recovery from AUD, there is evidence that active partner coping predicts positive outcomes. Specific types of active partner coping that support successful change include (a) decreasing negative or controlling behaviors that serve as antecedents to drinking; (b) increasing supportive and problem-solving communication; (c) reinforcing positive behavior change by the partner with an alcohol problem; (d) increasing shared positive activities; and (e) reducing family member drinking behavior to support changes in the drinking of the person with AUD.^20^

Families also may make recovery more difficult. For example, individuals with AUD perceive relationship problems as significant relapse precipitants,^21^ and believing that one’s partner also has AUD predicts poorer drinking outcomes compared to individuals who did not believe that their partners have AUD.^22^ Specific family behaviors associated with relapse include negative attitudes, emotional responding, and low levels of distress tolerance.^19^

## THE ROLE OF FAMILY-INVOLVED TREATMENT IN FOSTERING RECOVERY

Knowledge of the impact of AUD on families has led to the development of family-engaged treatments. Considerable research has focused on the development and testing of these family-engaged treatments to foster recovery from AUD. These treatments have focused on the role of the family in the initiation of help seeking, initiation of change, and maintenance of long-term change. The following sections describe and review treatments for affected family members in their own right, and as a way to help effect change in the identified individual with AUD. This is then followed by a review of the array of interventions influenced by cognitive behavioral therapy (CBT) and family systems models. Table 1 provides a summary of key elements in each of the treatments reviewed.

### Treatments for Affected Family Members

The 5-Step Method, a systematic intervention based on the SSCS model, is designed to help families cope more effectively with the AUD of a family member. The focus of the intervention is on the families in their own right, rather than on the relationship between family behaviors and outcomes for the person with AUD. The 5-Step Method helps families explore sources of stress and strain in their lives, provides psychoeducation about the SSCS model, helps them identify effective ways of coping with these sources of stress, assists them in identifying sources of social support for themselves, and assists with other needs that family members might have. The 5-Step Method has been tested with families in primary care as well as specialty care settings, with results supporting the effectiveness of the approach in reducing family-related harm in terms of both physical and psychological symptoms.^23^

Two treatments focus on providing family members with skills to help a family member to seek AUD treatment. CRAFT helps concerned family members to change contingencies for drinking by decreasing behaviors that protect the drinker from naturally occurring consequences of drinking, increasing positive family responses to changes in drinking, learning self-care and protection from intimate partner violence, and learning how to communicate positive requests for change and/or help seeking.^17^ Compared to Al-Anon, CRAFT results in significantly greater rates of help seeking, and comparable rates of improvement in family members’ depression and anxiety. The ARISE method (A Relational Intervention Sequence for Engagement) provides a series of steps that family members may use to encourage their loved one to seek treatment; ARISE also is effective in encouraging persons with AUD to seek treatment.^24^ In addition to treatments for the affected family member alone, there are several treatment models and approaches that involve both the affected family members and the individual with AUD. Treatments with strong empirical support have drawn largely from cognitive behavioral and family systems concepts; the following sections review these approaches.

### Cognitive Behavioral Approaches

Cognitive behavioral therapy (CBT) approaches view alcohol use as a learned behavior, cued by environmental stimuli and maintained by the positive consequences of alcohol use. Family-engaged CBT approaches view family behaviors as potential cues for drinking, as providing positive consequences of drinking, and as having the potential to provide positive consequences for changes in drinking behavior.

Adding partner-assisted components to individual treatment might involve partners assisting the person with AUD with accurate self-monitoring of alcohol intake and contributing to functional analysis of drinking patterns to help identify high-risk situations in which craving and alcohol consumption are likely to present a challenge. Psychoeducation is also common to help the partner more clearly understand the treatment needs and program of recovery for the person with AUD. Partner involvement might provide additional benefits such as helping the partner without AUD to develop new skills to reinforce changes in drinking and minimize behaviors that might contribute to maladaptive couple and family interactions. One recent study exemplifying this approach found support for integrating romantic partners into individual motivational interviewing interventions to improve individual AUD outcomes.^25^,^26^

**Figure 1:**
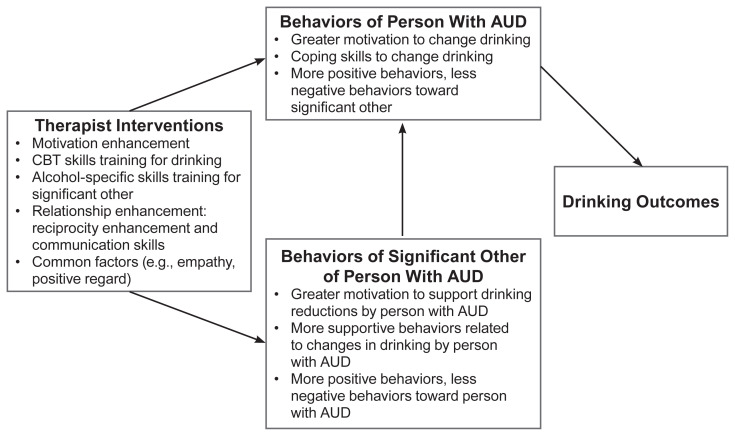
Hypothesized mechanisms of change in Alcohol Behavioral Couple Therapy. *Note:* AUD, alcohol use disorder; CBT, cognitive behavioral therapy.

Several manual-guided conjoint couple therapies incorporate cognitive behavioral techniques that have proven useful in individual treatments along with couple-focused interventions. One such modality with strong empirical support for both men and women with AUD is Alcohol Behavioral Couple Therapy (ABCT).^20^ ABCT is a 12-week, cognitive behavioral treatment that has demonstrated efficacy in reducing alcohol consumption, enhancing relationship functioning, and improving partners’ skills to facilitate reductions in drinking.^27^ Core components of ABCT include (a) CBT interventions to help the person with AUD change his or her drinking, (b) psychoeducation for the intimate partner to learn how to support changes in the behavior of their partner with AUD and to decrease behaviors that might serve as triggers for drinking, (c) interventions to teach the couple how to deal more effectively with drinking situations and drinking urges, (d) behavioral couple therapy interventions to increase positive interactions and improve communication skills, and (e) couple-focused relapse prevention. Figure 1 summarizes the hypothesized mechanisms by which ABCT impacts drinking outcomes. Recent ABCT literature indicates a strong association between partner participation in treatment and AUD outcomes. Reductions in drinking have been associated with increases in partner coping, conflict resolution skills, relationship satisfaction, and support behaviors.^28^ Greater relationship quality before treatment predicted abstinence and alcohol consumption posttreatment.^29^ Greater relationship satisfaction also is associated with fewer drinking urges and greater reduction in drinking urges during ABCT.^30^ One notable strength of ABCT is that it results in positive outcomes for couples presenting with poor relationship functioning and high levels of psychiatric comorbidity, and it is equipped to treat couples in which one or both partners have AUD.^27^

A second well-researched approach to couple-involved therapy is behavioral couples therapy (BCT) for AUD and other SUD.^31^ BCT is a 12- to 20-session intervention that lasts 3 to 6 months. The core components of BCT include (a) a daily “recovery contract” to encourage abstinence from substance use, (b) interventions to increase positive couple behaviors, and (c) training in behavioral communication skills. Participants with SUD also complete weekly urine drug screens, and progress is monitored in a calendar-assisted approach (similar to the Timeline Follow-Back procedure).^32^

Like ABCT, BCT is suitable to implement alongside 12-step groups such as Alcoholics Anonymous (https://aa.org/) and individual AUD treatments. Data from randomized controlled trials suggest that BCT has excellent feasibility, participant acceptability, and efficacy.^33^,^34^,^35^ BCT also has the ability to reduce maladaptive couple conflict behaviors such as intimate partner violence^36^ and has been tested for use among military veterans with positive outcomes^37^ and with couples in which both partners have AUD.^38^ However, findings from one recent trial indicate that a group adaptation to BCT to treat multiple couples simultaneously did not perform as well as when couples were treated separately.^39^

Brief family-involved treatment (B-FIT) is a three-session intervention that aims to improve family functioning, increase family-related incentives associated with reduced alcohol consumption, and implement proven techniques for family treatment of AUD to achieve and maintain long-term abstinence.^40^ Specifically, B-FIT incorporates adaptations such as (a) involving any concerned family member rather than romantic partners only, (b) implementation within a patient’s multifaceted program of recovery, (c) targeting the key components of ABCT in an accelerated manner, and (d) leveraging behavioral contracting techniques to increase treatment efficiency.^41^ B-FIT was recently examined in a pilot randomized controlled trial (*N* = 35 couples) with promising outcomes.^42^

### Family Systems Approaches

Treatment models based in family systems theory assume that the actions of individual family members affect all other members of the family, and that families have typical and repetitive ways of interacting that maintain dysfunctional behavior patterns of the family as a whole and of individuals within the family. Thus, these models focus on change in the structure and functioning of the family to effect change in dysfunctional behaviors, such as alcohol or drug use, in individual family members. Three major approaches in family systems therapy have evidence supporting their efficacy and should be noted, although most of the controlled trials of these treatments have been conducted primarily with adolescents with AUD or other SUD.

Brief strategic family therapy (BSFT) combines interventions from structural and strategic family therapies and assumes that substance use as well as other behavioral problems are symptoms of family dysfunction. Thus, the treatment focuses on influencing maladaptive patterns of family interaction, alliances, boundaries, and scapegoating of individual family members. Data reported from multiple studies support that BSFT is efficacious in decreasing adolescent substance use a year after treatment, that changes in family functioning mediate the relationship between BSFT and outcomes, and that parents receiving BSFT also decreased their drinking after treatment.^43^

Multidimensional family therapy (MDFT) views adolescent problems as multidimensional and addresses factors on multiple levels (i.e., individual, family, environment) that may be contributing to the adolescent’s problem behaviors. The treatment involves establishing multiple relationships between the therapist and the adolescent, family, and other systems, and it uses a range of interventions to restructure family and individual functioning. Data suggest that MDFT is more effective than comparison treatments,^43^ although it is more costly to deliver. However, when the associated costs of delinquency are considered, the cost-effectiveness of MDFT is comparable to cognitive behavioral interventions.^44^

Multisystemic therapy (MST), developed as a family intervention for youth involved with the juvenile justice system, intervenes in multiple systems, including the individual, family, school, peer, and community. The primary focus of MST has been on antisocial behaviors, but data also suggest that, compared to community treatment as usual, MST leads to positive substance use outcomes.^43^ Combined with interventions to strengthen families with parental AUD and child maltreatment, MST has been found to decrease child negative symptoms, parental substance abuse, and instances of child maltreatment.^45^

### Summary of Family-Involved Treatments

Efficacious treatments drawn from cognitive behavioral and family systems theories have been developed both for family members alone and for family members together with the individual with AUD. Most controlled trials of these treatments have compared either the family-involved treatment to treatment without the family member, or variations on the specific treatment (e.g., ABCT with or without involvement in Alcoholics Anonymous). Thus, the research literature to date does not provide guidance to clinicians about selecting a treatment from among those with empirical support.

## SPECIFIC POPULATIONS

A great deal has been learned to date regarding efficacious family and couple treatment models. However, the empirical literature is also clear that AUD is a condition characterized by a great deal of heterogeneity in etiology, course, and factors influencing treatment outcomes. The following section describes treatment considerations for populations that might require tailored treatment considerations and adaptations to optimize outcomes.

### Military and Veteran Families

Rates of hazardous and harmful alcohol use and AUD are high among active duty military and veteran populations. Compared to age- and sex-matched civilian samples, both women and men in active duty and veteran populations consume alcohol more frequently and heavily as well as incur a nearly fivefold greater risk for experiencing harmful alcohol-related health outcomes and developing AUD. Toward the goal of improving the health of the U.S. armed forces, their partners, and their families, emerging research has examined existing or adapted behavioral treatment approaches to determine their appropriateness in military and veteran populations, including couple therapy and treatment for families of veterans with AUD. For example, one recent open-label trial examined an adaptation of ABCT for returning military veterans (*N* = 44 couples).^46^ This study utilized a 15-session format and incorporated relevant topics for combat veterans, including intimate partner violence, depression, post-traumatic stress disorder (PTSD), and traumatic brain injury, which are all known to co-occur at high rates with heavy drinking and to affect military populations disproportionately. Similarly, BCT has demonstrated efficacy among veterans with AUD and co-occurring PTSD. More recently, a novel integrated approach that combines BCT with Cognitive Behavioral Couples Therapy for PTSD (Couple Treatment for AUD and PTSD) has shown promise in a preliminary open-label pilot study (*N* = 13 couples).^37^ Given that military culture places heavy emphasis on marriage and family, this population is ripe with opportunities to advance dyadic alcohol research to better understand how veteran and active duty families cope with and encourage recovery from AUD, and how the family as a whole changes as the person with AUD recovers. In addition, more attention is needed to address the unique challenges to implementing dyadic treatment in active duty and veteran treatment settings (e.g., frequent relocations, extended deployments).

### Women

Women with AUD experience different challenges than men with AUD in general and particularly in terms of intimate relationships. Data from longitudinal research suggest that husbands’ drinking patterns prior to marriage strongly predict women’s drinking in the first year of marriage, and male partners of women with AUD are more likely than wives of men with AUD to have AUD as well.^47^ Women with AUD see relationship problems and the male partner’s drinking as important antecedents to relapse, and they use alcohol to cope with relationship problems. Male partners of women with AUD tend to avoid confrontation as a way to cope with the woman’s drinking.^48^

The efficacy of ABCT and BCT has been tested with women with AUD and their male partners.^47^,^49^,^50^ In all three studies, ABCT or BCT led to better alcohol use outcomes for the women compared to the control condition. McCrady and colleagues also found that women who entered treatment with higher levels of relationship distress and women who presented with another clinical and personality disorders had greater improvements in drinking with BCT than individual therapy.^47^ However, if given the choice, women with AUD prefer individual rather than conjoint therapy, citing as reasons their desire to work on individual problems, their perception of a lack of support from their partner, and logistical challenges to attending treatment together.^51^

### Racial and Ethnic Minority Populations

Race and ethnicity play a significant role in family and couple relationship structure and functioning for many persons with AUD, thereby influencing the complex role of the family in AUD treatment seeking and recovery trajectories. To develop the knowledge base regarding the mechanisms by which race and ethnicity influence AUD recovery in families, dyadic AUD research must improve diversity within samples and must focus on treatment development adaptations for specific diverse populations. The existing literature demonstrates that substantial differences exist in alcohol consumption patterns, etiology, and risk factors associated with developing AUD as well as treatment engagement and outcomes in different racial and ethnic groups.^52^ Racially and ethnically diverse minority populations are persistently underrepresented as participants in randomized controlled trials focused on alcohol use. AUD research on families and couples faces a similar constraint that currently limits the generalizability of current findings.

Cultural constructs and institutional marginalization are likely to impact AUD recovery among racial and ethnic minority groups in varying ways. Furthermore, the complex intersectionality of various cultural and institutional factors is likely to influence drinking and recovery. Among other factors, gender roles, socioeconomic status, health care access, employment status, immigration status, involvement with the criminal justice system, religion, and language barriers are likely to manifest in separate but overlapping ways among families who belong to racial and ethnic minority groups.^53^,^54^ Some research suggests that acculturation and “traditional” family structures more often identified in non-White, non-Hispanic families might prevent the onset of AUD and facilitate effective treatment seeking and change in racial and ethnic minority groups.^52^,^55^ Conversely, stigma and cultural beliefs related to AUD and help seeking, as well as couple and family therapy specifically, might negatively influence AUD recovery processes for some members of racial and ethnic minority groups. However, these mechanisms have not been well tested in the context of couple or family treatment for AUD.

### Socioeconomic Status

Socioeconomic status (SES) is defined by many variables, including educational access and level, occupational status, housing access, neighborhood factors, and income.^56^ Although AUD occurs among individuals and families from all socioeconomic backgrounds, the direct association between socioeconomic status, AUD, and alcohol-related harms is complex.^57^ However, research indicates that families with lower SES (based on factors such as income and educational level) might incur increased negative physical and mental health sequelae of AUD, encounter barriers to accessing treatment, and confront more barriers to successful treatment outcomes, compared to families with higher SES.^53^,^54^,^57^,^58^ Minimal research has been conducted regarding socioeconomic barriers to accessing couple therapy for AUD specifically; thus, research is necessary to identify potential socioeconomic disparities and pathways to mitigating them. One study of access to general couple therapy was conducted among couples living in neighborhoods with at least 30% of households below the poverty threshold. Results showed that when couples in this sample obtained access to treatment, they utilized couple therapy services and derived positive gains.^59^ Thus, research is needed to better understand AUD recovery among families with different socioeconomic advantages or disadvantages. Studies investigating effective methods to increase access to low-cost treatment options—including those with technological adaptations to increase treatment availability—are warranted. Leveraging existing study data and using qualitative data collection techniques to identify barriers and methods to overcoming barriers are also needed.

### Sexual and Gender Minority Populations

Individuals identifying as sexual and gender minorities are more likely to consume alcohol and have higher rates of AUD than individuals identifying as heterosexual.^60^ Some accruing research suggests connections between alcohol use, AUD, and relationship functioning in this population. For example, in same-sex male couples, poorer relationship functioning appears related to higher rates of alcohol problems;^60^ in same-sex female couples, higher levels of verbal aggression and physical violence are associated with higher levels of alcohol use;^61^ and differences in alcohol use in same-sex female couples are associated with poorer relationship functioning (e.g., poor conflict resolution, poor satisfaction).^62^ However, research on intimate or family relationships and recovery in sexual minority groups is very limited. One qualitative study of gay men in recovery examined familial and other social network influences on recovery.^63^ Family and other social network factors cited as important to their recovery included acceptance of their sexual orientation and a sense of social connectedness. Conversely, although the men indicated that they continued to look to their families for support, many continued to experience family rejection of their sexual orientation and perceived this as a stressor that made recovery more difficult.

### Engaging Communities in AUD Treatment

A crucial shift emerging in the AUD treatment community is the recognition that treatment approaches need to be adapted to accommodate families from diverse backgrounds, rather than expecting individuals and families to adapt to current treatment methods. To achieve this goal, research is needed on how to modify current approaches to reduce pervasive barriers to identification of AUD, how to develop evidence-supported approaches to treatment access and engagement relevant to diverse populations, and how to include diverse communities in the scientific process (as both participants and investigators). Increasing partnerships between research and AUD provider teams with health systems and community representatives serving racial and ethnic minority families, families with limited economic resources, and sexual minority populations might reveal pathways to achieve this goal. Community-based participatory research is an approach that provides one framework for developing research through true community partnerships.^64^

## FUTURE DIRECTIONS FOR RESEARCH

During the past several decades, the empirical literature has expanded significantly to develop a critical foundation of knowledge and advance the implementation of family and couples-based approaches to AUD treatment. This section reviews promising areas for future research to further advance the state of the science in this area and to inform clinical best practices to optimize the AUD recovery process by incorporating family members.

### Understanding Couple and Family Support in Recovery

Data are limited on the role of couple and family support in AUD recovery processes outside of treatment; most of our knowledge to date has come from clinical trials of specific couple- or family-involved treatments or from studies using patients in treatment programs. A related question that warrants attention in the literature is learning about the circumstances under which partners and family members are well suited versus possibly inappropriate for conjoint therapies. Clinical guidelines for couple therapy for AUD suggest that conjoint therapy should not be attempted for couples with intimate partner violence that has resulted in physical harm or fear of retaliation or for couples in which one partner is planning to leave the relationship.^20^ Gaining a clearer understanding of the specific couple and family behaviors that support or are detrimental in AUD recovery, as well as the mechanisms by which these behaviors influence AUD recovery, is crucial to improve alcohol prevention and treatment efforts. For example, studies examining family-specific interactive behaviors that increase or mitigate known precipitants to drinking and relapse risk, such as heightened craving, are warranted. Similarly, this literature can be improved by examining thoughts, behaviors, and emotions that acutely predict both positive and negative AUD treatment outcomes, including those that occur within and between treatment sessions.

### Exploring Partner and Family Integration in Recovery-Oriented Systems of Care

Although the majority of the current review has focused on manual-guided and single-episode treatment approaches, it is widely recognized that more integrated and sustainable resources often are warranted to initiate and maintain AUD recovery across populations. During the last two decades, research focused on recovery-oriented systems of care (ROSC) has demonstrated positive findings.^65^–^69^ ROSC is defined as “networks of organizations, agencies, and community members that coordinate a wide spectrum of services to prevent, intervene in, and treat substance use problems and disorder.”^65^ Identifying pathways to integrate partners and family members, where appropriate, into ROSC models holds promise, but has not been investigated thoroughly. Future research directed at examining facilitators and barriers—at the patient, provider, and system levels—to inviting family members into AUD treatment under this model is necessary. For example, some individuals engaged in ROSC might be facing obstacles such as homelessness or incarceration that might make it more challenging to identify and engage a supportive peer, partner, or family member. Under these circumstances, an adjunctive approach to developing or strengthening nonfamilial social support relationships could be explored. It also is possible that improved training in existing couple and family theory and treatment modalities could facilitate greater accessibility and treatment outcomes.

### Role of Partners and Family in AUD Resilience

The existing literature can be improved by developing a better understanding of couple- and family-level factors promoting AUD resilience, with a particular focus on individuals, couples, and families who choose to change their drinking behaviors without engaging formal treatment resources. Recent literature has begun to expand the knowledge base regarding individual-level behavioral and neurobiological factors associated with greater likelihood of sustained recovery. However, less research has focused on the specific roles of partner and family members in changing drinking behaviors, neurobiological functioning associated with recovery-related cognitions and behaviors, and recovery when formal treatments are not engaged.^70^–^72^ Extending this area of the literature might be particularly useful for diverse populations with disproportionate risk for developing AUD or disparities and barriers to accessing formal or traditional AUD treatment resources.^73^,^74^

### Specific Populations

Couples and families from diverse backgrounds differ in their values, the structure and functioning of the families, gender roles within these relationships, how family members influence and support each other, and the role of alcohol use and AUD in the family. Although awareness of diversity in family functioning among different racial and ethnic groups, socioeconomically challenged populations, sexual and gender minorities, and veteran populations is increasing, the specific associations between alcohol use, AUD, family functioning, and AUD recovery have not been studied. Future research needs to focus on developing a more nuanced understanding of family structure and function around AUD in diverse populations to develop effective family-engaged treatments and dissemination of knowledge of effective practices to support recovery for these populations.

### Expanding Couple and Family Treatment for AUD

#### Technology

One new direction for dyadic AUD treatment is the integration of existing and emerging modalities with electronic and technologically based adaptations (e.g., smartphone/online access, e-health [electronic health], m-health [mobile health]). Such adaptations hold promise to facilitate treatment access and engagement, enable accuracy in assessment, reduce participant burden, and streamline delivery of treatment content.

Among individual participants, technology-assisted and fully technology-based interventions are rapidly proliferating in the alcohol field. Technology-based approaches have proven utility to inform novel treatment development efforts, and they focus existing interventions on key components that are most likely to yield significant impacts on alcohol-related cognitions and behavior. Studies conducted among individuals consistently find that technology-assisted modalities are highly feasible and acceptable among participants. They show promise to increase participant access, engagement, and outcomes; to improve reach and cost-effectiveness; and ultimately to provide a viable AUD treatment option for individuals in a variety of populations.^75^,^76^ An emerging body of literature is examining technology-based, e-health, or mobile interventions for couples with AUD. Findings from the limited emerging literature on technology-based couple interventions are encouraging. For example, one recent study tested a mobile support system to facilitate family communication among families affected by AUD (*N* = 9).^77^ Another study examined the feasibility and acceptability of a novel, four-session, web-based AUD intervention for military and veteran couples (*N* = 12) with promising outcomes.^78^ As remote telehealth (e.g., using telephone and/or videoconferencing) approaches are evolving in the AUD treatment field, an emerging literature suggests that telehealth implementation of couple and family therapy is also feasible and acceptable.^79^ Recent research on a brief, in-person, home-based couple intervention found positive results for enhancing accessibility and efficacy.^80^ Creating a home-based family telehealth intervention model of recovery has the potential to improve treatment access for individuals in AUD recovery and their partners and families.

A recently completed Small Business Innovation Research Phase 1 development project created a novel e-health intervention for families to reduce driving while intoxicated (DWI) and DWI recidivism.^81^ The intervention, B-SMART, was designed to help reduce risk for DWI reoffending by leveraging environmental support (e.g., family support) known to reinforce and thus increase the likelihood of alcohol abstinence and simultaneously reduce harmful drinking outcomes. Participants (*N* = 32) were family members of individuals with a recent DWI arrest and an interlocking ignition device installed on their vehicle, who rated the useability of the smartphone app. A Small Business Technology Transfer Phase 2 grant is underway to develop additional intervention modules and to conduct a randomized trial of the efficacy of the intervention.^82^ Overall, a great deal more research is needed to adapt existing dyadic AUD treatment modalities to incorporate technology such as mobile or online assessment monitoring, telehealth sessions, or self-guided online interventions.

#### Pharmacological treatment of AUD for couples and families

Combining pharmacological interventions with evidence-based behavioral treatments has the potential to optimize and sustain AUD treatment outcomes.^83^–^85^ However, few studies have examined the role of pharmacological interventions in trials of conjoint or family treatments for AUD. Research aimed at examining the role of medication utilization and compliance in dyadic and family modalities is needed. More specifically, medication-enhanced psychotherapy for AUD, in which medications and behavioral interventions are designed to work synergistically within or between sessions, is a promising new direction for couples. As new medications for AUD are being developed specifically with the goal of targeting brain stress and social reward systems (e.g., intervening in the withdrawal/negative affect and preoccupation/anticipation stages of AUD), medications to simultaneously maximize AUD outcomes and enhance relationship functioning could optimize AUD and relationship functioning outcomes among couples.^86^–^91^ One such medication, intranasal oxytocin, is currently being examined among couples with AUD for that purpose.^92^ Phase II trials of 3,4-methylenedioxymethamphetamine (MDMA) also are being conducted for a variety of psychiatric conditions, including among couples, and could hold promise to augment dyadic intervention for AUD.^93^

#### Neurobiological underpinnings of AUD

Current AUD research has a heavy emphasis on understanding the neurobiological and behavioral underpinnings of AUD and interactions between them. Such approaches have proven utility in novel treatment development efforts. However, advanced neurobiological measures and techniques, which have proven useful in treatment development efforts with individuals, have not yet been applied to couples. For example, clinically relevant AUD biomarkers are rarely examined in epidemiological or treatment research with couples. Similarly, although functional magnetic resonance neuroimaging is widely used in laboratory and treatment research in the alcohol field, there is a scarcity of literature examining resting state or task-related neural functioning in romantic couples. Some novel directions include hyperscanning, in which two participants are scanned simultaneously in response to shared stimuli, and adapting imaging paradigms to address relational behaviors relevant to AUD.^94^,^95^ Preliminary evidence from a small sample of couples with relationship distress and substance misuse suggests that intimate partner violence in the relationship might exacerbate neural stress responses associated with couple conflict cues.^96^ When applied to either mechanistic or treatment development efforts, this emerging line of literature might help to develop neural prognostic and diagnostic indicators of positive AUD treatment outcomes, risk for AUD relapse, and short- and long-term correlates of AUD relapse risk.

Another area of potential for future research is applying the existing literature on dyadic physiological and neuroendocrine co-regulation to the alcohol field, an effort that has begun but needs to be extended. Data collected from samples of couples experiencing relationship distress and who enrolled in treatment trials for problems other than AUD indicate that discordant dyadic autonomic dysregulation is associated with acute and more severe couple conflict,^97^ whereas synchrony in autonomic functioning is indicative of constructive couple therapy processes such as working alliance and improved health outcomes.^98^ As biofeedback intervention approaches continue to evolve in the AUD field, these emerging data can help to inform the development and refinement of remote and in-person dyadic biofeedback to support recovery efforts among families affected by AUD.

#### Involvement of partners and family members in AUD therapies in the context of co-occurring mental health conditions

Identifying pathways to successfully treat AUD and co-occurring conditions among individual participants remains an area of intense scientific inquiry. However, far less attention has been dedicated to understanding how partners and family members might contribute to adjunct or conjoint therapies. One preliminary pilot study found promising feasibility and acceptability outcomes when examining a novel integrated approach that combines BCT with Cognitive Behavioral Couples Therapy^99^ for PTSD (*N* = 13 couples).^37^ Research also suggests that ABCT is more efficacious than individual CBT for women with AUD and co-occurring clinical and personality disorders.^47^ A great deal more research is needed to identify dyadic pathways to treating AUD and commonly co-occurring conditions such as PTSD and depression.

#### Dissemination and implementation

Despite the abundance of rigorously conducted studies and findings supporting the efficacy of dyadic AUD treatment, evidence-based couple and family therapies are rarely applied in frontline treatment settings. Literature identifying barriers to provider uptake and patient utilization is also limited. The scant data available suggest that a lack of familiarity with modalities such as BCT among treatment providers and administrators of treatment clinics are among the most commonly cited challenges.^100^ Additional challenges include (a) logistical and time-related barriers to scheduling sessions with both members of a couple; (b) a lack of clarity regarding insurance reimbursements available for couple therapies (and whether reimbursements are greater than for individual sessions); (c) lack of formal training in couples therapies for AUD; and (d) perceived increase in the difficulty of implementing dyadic treatment compared to treating individuals with AUD.^100^ As a result, dissemination and implementation efforts are needed to identify more clearly provider and administrative barriers to uptake across various treatment settings (e.g., community clinics, Veterans Affairs clinics, academically affiliated clinics), to develop accessible provider education models, and ultimately to develop a more robust and diverse pipeline of capable and confident providers.

The majority of individuals with AUD who change successfully do so on their own, without any formal treatment.^101^ As knowledge accrues about the most effective ways for families to motivate persons with AUD to change and to support change efforts, models to disseminate this knowledge in provider training programs and outside of treatment settings are needed. Community-based studies of these dissemination efforts also are needed to advance provider education and training efforts and to promote utilization of the full scope of couple and family treatments for AUD that are both available and efficacious.

### Mechanisms of Treatment Response

Although efficacious couple and family treatments for AUD have been developed and tested, knowledge regarding behavioral mechanisms of action underlying treatment response largely remains untested. It is possible that both individual and relational mechanisms specific to family and couple interactions might facilitate improved treatment outcomes, maintenance of recovery programs and sobriety, and long-term health. Thus, studies examining the mechanisms of action underlying effective couple and family treatments for AUD—as well as secondary analyses of extant data sets and studies combining data sets from multiple randomized controlled trials—are warranted. One avenue to addressing this gap in the literature is the use of observational coding schemes to examine within-session behaviors indicative of treatment response. A recent study examined the association between pronoun utilization (i.e., “I” versus “we”) within ABCT sessions and found that greater “we” language utilization was associated with greater alcohol abstinence at end of treatment and follow-up.^102^ Recent analyses based on coding of within-session language in ABCT sessions have found that contemptuousness by individuals with AUD toward their partners predicts poorer drinking outcomes^103^ and that within an ABCT treatment session there is a complex interaction among client and partner change language and positive and negative relationship behaviors.^104^ This line of research can be expanded to further improve our understanding of within-session behaviors relevant to AUD recovery among couples and families, given that several reliable and valid observational coding systems (i.e., the Rapid Marital Interaction Coding System [RMICS]; System for Coding Couple Interaction in Therapy–Alcohol [SCCIT-A]) have been developed and are widely used among couples in laboratory settings.

One specific mechanistic aspect of this literature that has not been thoroughly explored is the role of specific conflict behaviors and dyadic processes (both adaptive and maladaptive) in influencing alcohol craving as well as risk for lapse and relapse in AUD. The daily process and micro-longitudinal research designs and methods that have proven essential to understand some individual and dyadic mechanisms linking alcohol with couple conflict behaviors, such as intimate partner violence, have not been extended to nonviolent dyadic processes and recovery-related cognitions and behaviors. This literature could be advanced through innovative intersections of multi-method approaches that link laboratory, neurobiological, and naturalistic data, such as incorporating traditional clinical trial designs with micro-longitudinal and remote assessment methods. Such data might be used to inform novel and accessible adjunct interventions and tailored treatment modifications to insulate people with AUD and their families from high-risk situations.

### Leveraging Representative Samples

Future large-scale and multisite studies examining nationally representative samples (such as the National Epidemiologic Survey on Alcohol and Related Conditions [NESARC] data set,^105^ etiological processes (such as the Adolescent Brain Cognitive Development study [ABCD]),^106^ and treatment development (such as the Combined Pharmacotherapies and Behavioral Interventions for Alcohol Dependence [COMBINE study])^107^ have the ability to leverage rich infrastructures and diverse resources, often in a longitudinal fashion, to measure dyadic and family functioning using reliable and valid measures. To date, measurement of partner- and family-related variables has been limited in existing efforts. Increased collaboration between investigators and treatment providers with dyadic and family expertise pertaining to AUD is warranted in future integrated and large-scale efforts. As brief and empirically sound measurement approaches become more widely available, such collaborative efforts have the potential to reduce existing silos between fields of expertise within the AUD research community and ultimately to provide critical new information to drive the AUD field forward.

## SUMMARY AND CONCLUSIONS

The existing literature suggests that families play a key role in motivating persons with AUD to recognize the need to change, providing support for change, and supporting long-term recovery and that AUD recovery is good for families. Most of our current knowledge, however, has come from studies of relatively small clinical samples or from treatment studies. The lack of community-based research, multisite randomized controlled trials, research on integration of partners and family members in recovery-oriented systems of care, conduct of AUD treatment-specific meta-analyses, and the exclusion of couple- and family-level variables in large-scale longitudinal studies of the onset and course of AUD remain important areas for future research. Similarly, the lack of research on the role of the family in AUD recovery in diverse populations is a major gap in the current literature.

The existing literature from treatment studies suggests that integrating partners and family members into AUD treatment is a highly effective way to maximize positive treatment outcomes and to facilitate long-term AUD recovery and health of individuals with AUD and their families. Several manual-guided approaches have proven efficacy, but efforts to improve provider education and increase uptake of evidence-supported couple- and family-based AUD treatment modalities are needed to improve access and maximize the reach of available interventions. Challenges also might emerge if social relationships are persistently strained, if it is not safe or appropriate to include partners and family members in these modalities, or if individuals with an alcohol problem are navigating additional challenges such as incarceration or homelessness that are likely to influence day-to-day social contact and implementation of currently available modalities. There is an abundance of new opportunities to integrate emerging novel scientific methods—such as multimodal, multidisciplinary assessment and intervention approaches—into research focused on couples and families with a family member with AUD. The literature also is clear that improved access to AUD treatments among diverse populations is needed. It is crucial to improve synergy between existing alcohol research and the treatment community as well as the vast population of individuals in need of AUD treatment and their partners and families. Progress toward meeting these goals can be facilitated through increased collaboration with community partners to develop culturally informed modifications to research inclusion, AUD assessment, and intervention. Increased collaboration between investigators, administrators, and clinical providers to maximize existing federal funding investments in couple and family AUD treatment and recovery processes also holds potential to reduce treatment barriers and improve long-term outcomes for couples and families.

## Figures and Tables

**Table 1:** Family Interventions for AUD

Intervention	Number of Sessions	Target Population	Key Interventions
5-Step Method^23^	Variable/as needed	Family members	Explore sources of stress/strain Provide psychoeducation Identify ways of coping Identify social supports Address other family needs
Community Reinforcement and Family Training (CRAFT)^17^	12 or more	Family members	Decrease behaviors protecting from negative consequences Increase self-care Increase positive responses to changes in drinking Enhance self-care Protect from domestic violence Enhance communication skills
A Relational Intervention Sequence for Engagement (ARISE)^24^	3 or more	Family members	Level 1: telephone coaching to invite person with AUD to a meeting Level 2: face-to-face coaching with family Level 3: coaching family to set limits and consequences
Significant Other engagement in Motivational Interviewing (SOMI)^26^	1	Couples	Single session of motivational interviewing Partner skills to enhance motivation to change drinking Partner skills to support drinking reductions
Alcohol Behavioral Couple Therapy (ABCT)^20^	12 (weekly)	Couples	Cognitive behavioral therapy interventions to change drinking Partner skills to support change Partner skills to decrease antecedents to drinking Couple skills to manage drinking situations Enhance positive couple interactions Enhance couple communication skills
Behavioral Couples Therapy (BCT)^31^	12–20 (weekly)	Couples	Implement daily recovery contract Enhance positive couple interactions Enhance couple communication skills
Brief Family-Involved Treatment (B-FIT)^41^	3 (weekly)	Family member and person with AUD	Increase positive interactions Implement recovery contract Enhance family communication skills
Brief Strategic Family Therapy (BSFT)^43^	12–16 (weekly)	Whole families	Influence maladaptive family interactions, alliances, and boundaries Decrease scapegoating
Multidimensional Family Therapy (MDFT)^44^	40–48 (twice weekly for 5 to 6 months)	Whole families	Develop multiple therapeutic alliances Restructure family functioning
Multisystemic Therapy (MST)^45^	Approximately 20	Whole families; youth involved with juvenile justice system	Individual treatment Family intervention School-based intervention Peer-based intervention Community-based intervention
